# Loss of *GRHL3* leads to TARC/CCL17-mediated keratinocyte proliferation in the epidermis

**DOI:** 10.1038/s41419-018-0901-6

**Published:** 2018-10-19

**Authors:** Stephen J. Goldie, Denny L. Cottle, Fiona H. Tan, Suraya Roslan, Seema Srivastava, Rhys Brady, Darren D. Partridge, Alana Auden, Ian M. Smyth, Stephen M. Jane, Sebastian Dworkin, Charbel Darido

**Affiliations:** 1 Department of Medicine, Monash University Central Clinical School, Prahran, VIC 3004 Australia; 2 Department of Surgery, Monash University Central Clinical School, Prahran, VIC 3004 Australia; 3 Biomedicine Discovery Institute (BDI), Monash University, Clayton, VIC 3800 Australia; 4 Department of Anatomy and Developmental Biology, Monash University, Clayton, VIC 3800 Australia; 5 Department of Biochemistry and Molecular Biology, Monash University, Clayton, VIC 3800 Australia; 6 Division of Cancer Research, Peter MacCallum Cancer Centre, Grattan Street, Melbourne, VIC 3000 Australia; 7 Department of Physiology, Anatomy and Microbiology, La Trobe University, Melbourne, VIC 3086 Australia; 8 Sir Peter MacCallum Department of Oncology, The University of Melbourne, Parkville, VIC 3052 Australia

## Abstract

Identifying soluble factors that influence epidermal integrity is critical for the development of preventative and therapeutic strategies for disorders such as ichthyosis, psoriasis, dermatitis and epidermal cancers. The transcription factor Grainyhead-like 3 (GRHL3) is essential for maintaining barrier integrity and preventing development of cutaneous squamous cell carcinoma (SCC); however, how loss of this factor, which in the skin is expressed exclusively within suprabasal epidermal layers triggers proliferation of basal keratinocytes, had thus far remained elusive. Our present study identifies thymus and activation-regulated chemokine (TARC) as a novel soluble chemokine mediator of keratinocyte proliferation following loss of *GRHL3*. Knockdown of *GRHL3* in human keratinocytes showed that of 42 cytokines examined, TARC was the only significantly upregulated chemokine. Mouse skin lacking *Grhl3* presented an inflammatory response with hallmarks of TARC activation, including heightened induction of blood clotting, increased infiltration of mast cells and pro-inflammatory T cells, increased expression of the pro-proliferative/pro-inflammatory markers CD3 and pSTAT3, and significantly elevated basal keratinocyte proliferation. Treatment of skin cultures lacking *Grhl3* with the broad spectrum anti-inflammatory 5-aminosalicylic acid (*5ASA*) partially restored epidermal differentiation, indicating that abnormal keratinocyte proliferation/differentiation balance is a key driver of barrier dysfunction following loss of *Grhl3*, and providing a promising therapeutic avenue in the treatment of *GRHL3*-mediated epidermal disorders.

## Introduction

The mammalian epidermis forms a physical barrier between the internal and external environment^1^. This barrier plays a critical role in preventing tissue dehydration and providing protection from a number of deleterious agents including microorganisms, ultra-violet radiation and mechanical insults^2^. The establishment and maintenance of the epidermis is controlled by genetic networks that regulate keratinocyte proliferation, differentiation and enzymatic activity; a prolonged imbalance to any of these processes can compromise barrier function and lead to abrogated barrier integrity^2^. Moreover, this breakdown of barrier can lead to numerous skin disorders such as psoriasis and atopic dermatitis (AD)^1,3–5^, whereby prolonged impairment can progress to hyperproliferative disorders, such as epidermal cancers. Inflammation in response to barrier dysfunction has also been found to further impair keratinocyte differentiation and increase disease severity in Harlequin Ichthyosis^6^. As such, the identification of genetic networks and cytokine pathways, which regulate and maintain epidermal homeostasis, are a promising avenue to treat these skin disorders.

Recent studies have demonstrated that *Grainyhead-like 3* (GRHL3), a conserved developmental transcription factor, is essential for epidermal differentiation and barrier formation^7^. *Grhl3*-null mice (*Grhl3*^–/–^) die shortly after birth due to excessive water loss, a result of compromised skin barrier formation. This can be attributed in part to decreased expression of cross-linking enzyme *Transglutaminase 1*, a direct transcriptional target of GRHL3, as well as a number of other genes essential for barrier formation^7–9^.

GRHL3 also plays an important role in tumour suppression; mice with conditional deletion of *Grhl3* in adult epidermis (subsequent to barrier formation) survive minor regression of the preformed barrier^10^, however, these mice develop spontaneous skin tumours when aged and show increased susceptibility to chemical-induced squamous cell carcinomas (SCC)^11,12^. Interestingly, GRHL3 also suppresses the proliferation of human keratinocytes and was shown to function as a major tumour suppressor against human SCC^11,12^. Additionally, results from a recent study suggest that a GRHL3-regulated epidermal barrier repair pathway suppresses immune-mediated epidermal hyperplasia^13^. Together, these studies indicate that GRHL3 plays a key role in maintaining the integrity of the epidermis. However, the identity of responsive factors implicated in the epidermal hyperplasia following GRHL3 loss, particularly soluble cytokines, has thus far remained elusive. Accordingly, the aim of the current study was to identify key chemokines, which may be contributing to the pathogenesis of barrier disruption, epidermal hyperplasia and SCC observed in *GRHL3*-deficient mice and humans.

## Materials and methods

### Experimental animals

*Grhl3*^–/–^ mice were as described previously^11^. All animal experimentation was performed under approval granted by the Animal Ethics Committees of either the Alfred and Monash Research and Education Precinct or Monash Animal Research Platform.

### shRNAs and lentiviral infection

The immortalised human keratinocytes HaCaT were obtained from ATCC and cultured in Dulbecco’s modified Eagle’s medium (DMEM) supplemented with 10% foetal calf serum (FCS), 4 mM l-Glutamine and 100 μg/ml penicillin/streptomycin (P/S) solution. HaCaT cells were infected with two lentivirus short hairpin RNA (shRNA) targeting GRHL3 (shRNA1 and shRNA2) and a non-targeting control empty vector (EV) shRNA as described previously^11^. Efficiency of GRHL3 knockdown (KD) was confirmed by quantitative PCR (qPCR) (Fig. S1A). HaCaT cells transduced with the lentivirus shRNA1 were subjected to genome editing using CRISPR-Cas9-mediated deletion. Single guide RNAs targeting TARC (sgTARC) was transduced in shRNA1 to generate the HaCaT sgTARC + shRNA1 cell line. To induce expression of the sgRNA, doxycyline (Dox) Hyclate (Sigma-Aldrich D9891) was dissolved in sterile water at a stock concentration of 10 mg/ml and added to the tissue culture medium at a final concentration of 1 μg/ml.

### Colony-forming assay

HaCaT-EV and shRNA1 were seeded at a density of 500 cells/well in 10 cm diameter dishes (*n* = 3). In all, 50% of conditioned medium (CM) in HaCaT-EV cells were replaced with filtered CM harvested from shRNA1 cells and vice versa, 50% of CM in shRNA1 cells were replaced with filtered CM from HaCaT-EV cells. Cells were cultured for 21 days before staining as described previously^11^. Plates were scanned and then digital images were quantified using ImageJ (version 1.50i; NIH, Bethesda, MD).

### Human cytokine array

CM was generated by incubation of confluent HaCaT-EV, shRNA1 and shRNA2 transduced cells for 24 h. Media were harvested and filtered through 22 μm filters prior to incubation with a human cytokine antibody array membrane (Abcam, Cambridge, UK). Samples were processed and exposed to radiographic film to reveal cytokine levels present in the media. Films were scanned and quantified using ImageJ (version 1.50i; NIH, Bethesda, MD). These levels were normalised to non-CM, then comparisons were made between the EV and shRNA samples. Statistical significance was determined using GraphPad Prism and paired Student’s *t*-tests comparing the EV sample with each shRNA individually. Positive and negative controls were performed and analysed as per manufacturer’s instructions.

### Blood clotting assay

Skin from E18.5 *Grhl3*^–/–^ and wild-type (WT) littermate embryo controls were added to falcon tubes with 200 μl blood from an adult female *Grhl3*^*+/–*^ mouse (*n* = 3). After 15 s, the tubes were inverted and placed onto absorbent paper.

### SEM of embryo back skin

Samples of E18.5 *Grhl3*^–/–^ and WT littermate embryo control skin were processed for scanning electron microscopy (SEM) imaging according to previously described protocols^14^.

### Grafts of Grhl3^–/–^ skin onto NSG mice

E18.5 *Grhl3*^–/–^ and WT littermate embryo control skin was surgically grafted to the backs of adult (12-week-old) non-obese diabetic (NOD)–severe combined immunodeficient (SCID) gamma (NSG) mice as described previously^15^. After 4 months, the skin was harvested and processed for immunostaining according to standard protocols^11^.

### Immunofluorescence, immunohistochemistry and antibodies

Antibodies were anti-γδT cells (1:100, Sapphire Bioscience), anti-CCL17/TARC B22H33L5 (700655, 1:20–50, Thermofisher Scientific-Invitrogen, Frederick, USA), anti-Periplakin (ab72422, 1:200, Abcam, Cambridge, UK), anti-CD3 clone SP7 (1:500, M3072, Spring Bioscience), anti-p-STAT3 (Tyr705) (1:200, 9145S CST), anti-Keratin 14 clone LL002 (ab7800, 1:500, Abcam, Cambridge, UK), anti-Ki-67 clone SP6 (ab16667, 1:500, Abcam, Cambridge, UK) and anti-Keratin 10 clone RKSE60 (sc-23877, 1:50, Santa Cruz Biotechnology, CA, USA). Primary antibodies were visualised using Alexa-488 and Alexa-555 fluorescent secondary antibodies (Invitrogen, Molecular Probes). All confocal imaging was performed using a Nikon A1R Confocal microscope. For immunohistochemistry, slides were dewaxed and brought to water. Antigen retrieval was performed on a Dako PT Link (Agilent Technologies, Malaysia), using Dako Target Retrieval Solution (Dako, USA), for 30 min at 98 °C. Staining was performed on a Dako AutoStainer Link 48 Instrument (Dako, USA). Endogenous peroxidase activity was blocked using Dako Peroxidase Block for 10 min (Dako, USA) and Dako serum-free protein block (Dako, Denmark) was then applied for 10 min. Primary antibodies were diluted in Antibody Diluent (Dako, Denmark) and applied for 60 min at room temperature. Goat anti-mouse or goat anti-rabbit Dako EnVision + secondary antibodies (Dako, Denmark) were then applied for 30 min at room temperature. The signal detection was done using DAB (Dako, USA) for 10 min. Finally, the sections were counterstained with Automation Hematoxylin (Dako, USA) for 5 min, then dehydrated, cleared and mounted with DPX. Washes were done with Dako Wash Buffer (Dako, USA). Slides were dried then scanned with an Aperio brightfield slide scanner and viewed with ImageScope software. The ImageJ Immunohistochemistry Image Analysis Toolbox was used to assess pixel intensity.

### Ex vivo whole-mount embryo skin culture

Embryos were harvested at E16.5 under sterile conditions and cultured in an ex vivo system. The mid to lower back skin was isolated and cut into left and right side halves and cultured dermis-side down on six-well chamber inserts (Costar Transwell Permeable supports CLS3450-24EA). One skin section of each matched pair was grown at 37 ° with 1.5 ml of DMEM supplemented with 10% FCS, P/S and l-Glutamine media added to the lower chamber while the other was grown in media containing either vehicle, 1 mM 5-aminosalicylic acid (5ASA/mesalamine) (Sigma-Aldrich PHR1060) or human recombinant TARC (recTARC) (Abcam ab9817) at a final concentration of 100 ng/ml. This forms an air–liquid interface with the dermis drawing media from the lower chamber while the epidermis remained dry. Respective media were changed after 48 h and skin cultures harvested after 96 h of culture. The skin was then cut from chamber inserts leaving membrane backing intact for support during 4 h of fixation with 4% Paraformaldehyde (PFA) at room temperature. Samples were finally stored in 70% ethanol and membrane backing removed before being processed for paraffin histology using standard methods^11^.

### Vector and sequence for CRISPR-Cas9-mediated deletion of TARC

Plasmids for the sgRNA construct pFgh1UTG (ID#70183) and the FUCas9Cherry (ID#70182) backbone were gifts from Marco Herold obtained from Addgene. The sgRNA sequence for TARC was designed using a web-based tool CHOPCHOP (http://chopchop.cbu.uib.no/). For the cloning of the sgRNA, 24-bp oligonucleotides containing the sgRNA sequence were custom synthesised (Bioneer Pacific). The oligonucleotides included a 4-bp overhang for the forward (TCCC) and complementary reverse (AAAC) to allow cloning into the *Bsmb*-I restriction site of the lentivirus MCS. The sequence of the sgRNA was: 5′-CTCGAGGGACCAATGTGGGCCGG-3′. Following sgRNA transduction, RNA was extracted using Trizol as per manufacturer's protocol and 1 μg of RNA was used for cDNA synthesis for qPCR analysis. Primer sequences for TARC qPCR were: forward 5′-GAGCCATTCCCCTTAGAAAG-3′ and reverse 5′-AGGCTTCAAGACCTCTCAAG-3′.

### Proliferation assay

Cell lines were plated at a density of 1 × 10^4^ per well with or without CM as indicated. The number of living cells was counted every 2 days for 8 days using a Haemocytometer with 0.4% Trypan blue dye as a cell stain. Counts were performed in duplicates and repeated three times.

## Results

### The pro-proliferative cytokine TARC is upregulated in keratinocytes following *GRHL3* disruption

**Fig. 1:**
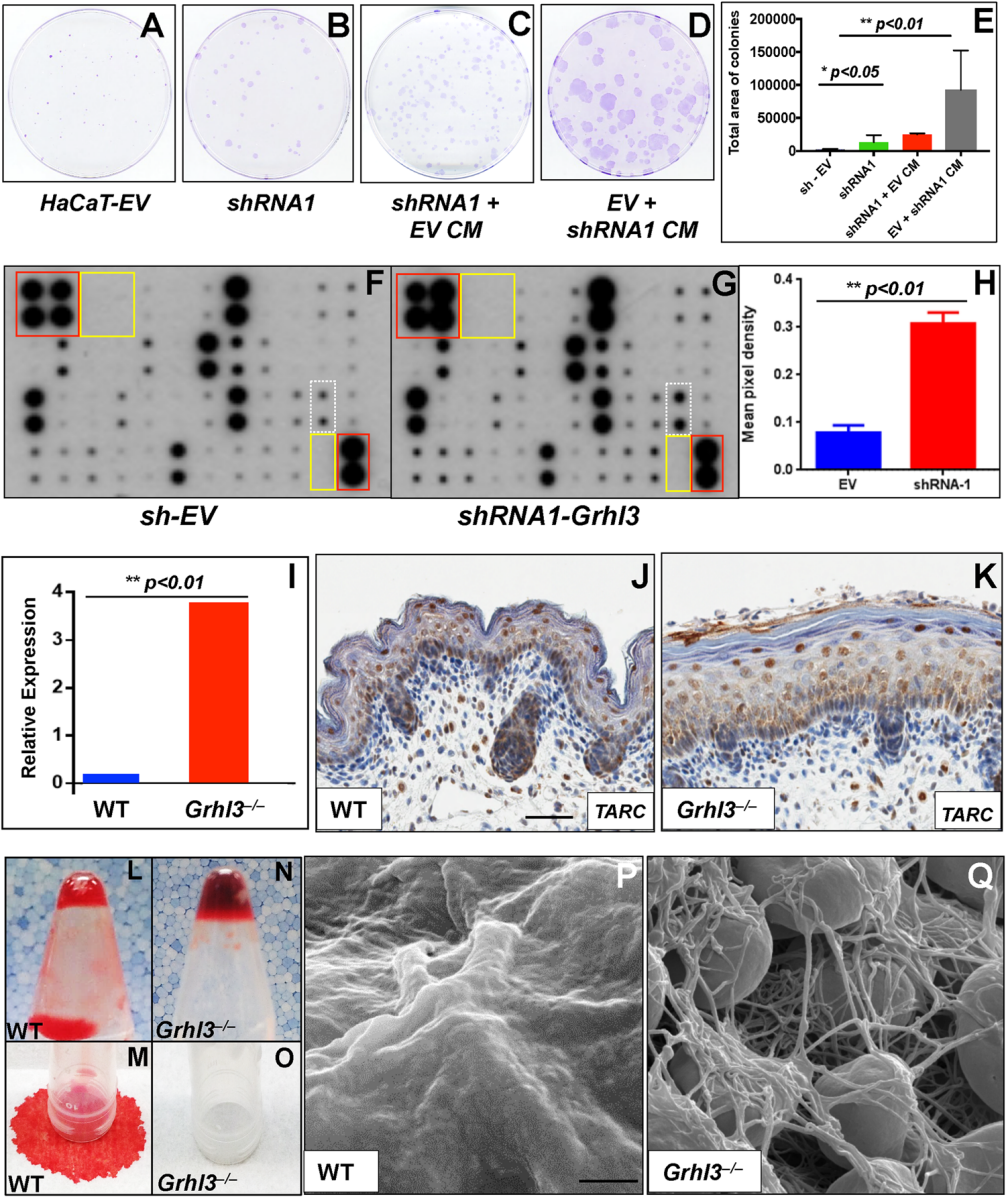
Loss of *GRHL3* leads to increased keratinocyte cell proliferation and elevated TARC expression. KD of *GRHL3* via shRNA (*shRNA1-GRHL3*) leads to increased colony size (**a,** **b**), relative to transduction with empty vector (*sh-EV*), albeit not increased colony number (data not shown) in the human epidermal keratinocyte cell line HaCaT. Conditioned medium (CM) from EV cultures did not stimulate growth of *shRNA1* cells (**c**). However, CM from *shRNA1* cultures stimulated significant growth of *EV* colonies (**d**), a finding confirmed by quantitation of total colony area per plate (**e**). Analysis of cytokine activity in CM collected from HaCaT cells transduced with *sh-EV* (**f**) or *shRNA1-GRHL3* (**g**) shows that the only cytokine that is significantly overexpressed (when quantitated by densitometric scanning; **h**) following *GRHL3* KD is TARC (dotted white box). Positive (red boxes) and negative controls (yellow boxes) are also shown. Q-RT-PCR quantitation of mRNA expression (**i**) of E18.5 back skin from WT and *Grhl3*^–/–^ embryos shows that TARC is significantly elevated in the skin of *Grhl3*^–/–^ embryos. Immunohistochemical analysis (**j**, **k**) shows predominant TARC expression in the nuclei of keratinocytes near or in the granular layer. Weaker basal TARC is also detected. Scale bars correspond to 50 μm. When WT embryonic skin is placed in a microtube with WT adult blood, (**l**, **m**), no clotting occurs, indicative of an absence of soluble clotting factors in the blood. However, when blood from the same adult animal is incubated with skin from *Grhl3*^–/–^ embryos (**n**, **o**), rapid clotting occurs. Ultrastructural analysis of blood on both WT (**p**) and *Grhl3*^*–/–*^ embryo skin (**q**) by scanning electron microscopy (SEM) shows no clotting of blood on WT skin, but the presence of strands of fibrin (indicative of clotting) is clearly seen on the *Grhl3*^–/–^ embryo skin. * and ** indicates *p* < 0.05 and *p* < 0.01, respectively. Scale bars correspond to 2 μm

In order to determine the role of *GRHL3* in keratinocyte growth, proliferation and putative paracrine signalling, we utilised two different shRNAs to KD *GRHL3* expression in the HaCaT human keratinocyte cell line, *shRNA1-Grhl3* and *shRNA2-Grhl3*, both as described previously^11^. We found that loss of *GRHL3* led to an increase in colony size and density (corresponding to an increased number of cells; Figs. 1a, b and Fig. S1B-D) without concomitant increase in colony number. Filtered CM from HaCaT cultures (EV) was added to *shRNA1-Grhl3* cells and demonstrated no significant increase in colony growth (Fig. 1c). Moreover, CM from *shRNA1-Grhl3* cultures was then added onto control HaCaT cells and stimulated significant growth of colonies (Figs. 1d, e). These data suggested that loss of *GRHL3* might lead to increased paracrine signalling through secretion of pro-proliferative factors within the culture dish. In order to test this hypothesis, we analysed the expression of 42 common cytokines (Fig. S2) in the culture media of both control and shRNA-*GRHL3* treated cells (Figs. 1f, g and Fig. S1E-F). Utilising a cut-off of cytokines, which were upregulated at least 1.5-fold following KD mediated by both anti-*GRHL3* shRNAs, we found that the only cytokine to be significantly upregulated (Fig. 1h, and Fig. S1G) was the thymus and activation-regulated chemokine (TARC), a paracrine signalling molecule known to be involved in promoting tumour proliferation, chemotaxis, inflammation and platelet activation, as well as being implicated in promoting the severity and progression of numerous skin pathologies. Interestingly, we generated a stable *shRNA1-Grhl3* HaCaT cell line with a Dox-inducible CRISPR-Cas9-mediated deletion of TARC (Fig. S3A). Cells with co-KD of *Grhl3* and *TARC* proliferated slower than cells with single *shRNA1-Grhl3* KD. In addition, CM from cells with co-KD of *GRHL3* and *TARC* did not promote HaCaT-EV proliferation to the same extent as the CM from *shRNA1-Grhl3* HaCaT cells (Fig. S3B). These data suggested that increased TARC expression following loss of *GRHL3* might contribute to epidermal keratinocyte hyperproliferation.

### TARC expression and function is upregulated in epidermis of mice lacking *Grhl3*

We next examined the expression of TARC in the back skin of WT and *Grhl3*^–/–^ mouse embryos at embryonic day (E) 18.5 of gestation, a time point at which we had previously noted an increase in epidermal proliferation in mice lacking *Grhl3*^11^. We found by reverse transcriptase-PCR (RT-PCR; Fig. 1i) that the expression of TARC was significantly increased within the skin of *Grhl3*^–/–^ embryonic mice. Examining TARC expression by immunohistochemistry in WT embryo skins showed peak TARC expression in differentiating suprabasal keratinocytes near the point of granular layer compaction and weaker TARC reactivity in less differentiated and basal cells with the exception of proliferative clusters such as hair germ. *Grhl3*^–/–^ embryonic skins exhibited a similar profile except the cell populations expressing TARC such as near granular layer keratinocytes were expanded (Figs. 1j, k), likely accounting for increases in TARC *mRNA*. Next, as TARC is a known mediator of platelet aggregation and clotting^16^, we placed a piece of epidermis from both WT and *Grhl3*^–/–^ E18.5 mouse embryos into a microfuge tube containing peripheral blood extracted from the (*Grhl3*^*+/–*^) mother. We found that blood containing WT skin flowed freely once the microfuge tube was inverted (Figs. 1l, m), whereas blood containing the skin from *Grhl3*^–/–^ mouse embryos was unable to flow down the walls of the tube following inversion, as it had clotted (Figs. 1n, o). Finally, in order to confirm clotting and to image the process in more detail, WT and *Grhl3*^–/–^ mouse embryos exposed to maternal blood on their skin, were processed and imaged using SEM (Figs. 1p, q). We found that on WT epidermis there was no evidence of adherent blood clotting, whereas an abundant meshwork of fibrin congregated around individual blood cells was clearly visible on the skin of *Grhl3*^–/–^ mouse embryos. Taken together, these data indicate that both TARC expression and clotting function are upregulated following loss of *Grhl3* in the skin.

### *Grhl3*^–/–^ embryo skin presents with barrier defects independent of a systemic immunomodulatory response

**Fig. 2:**
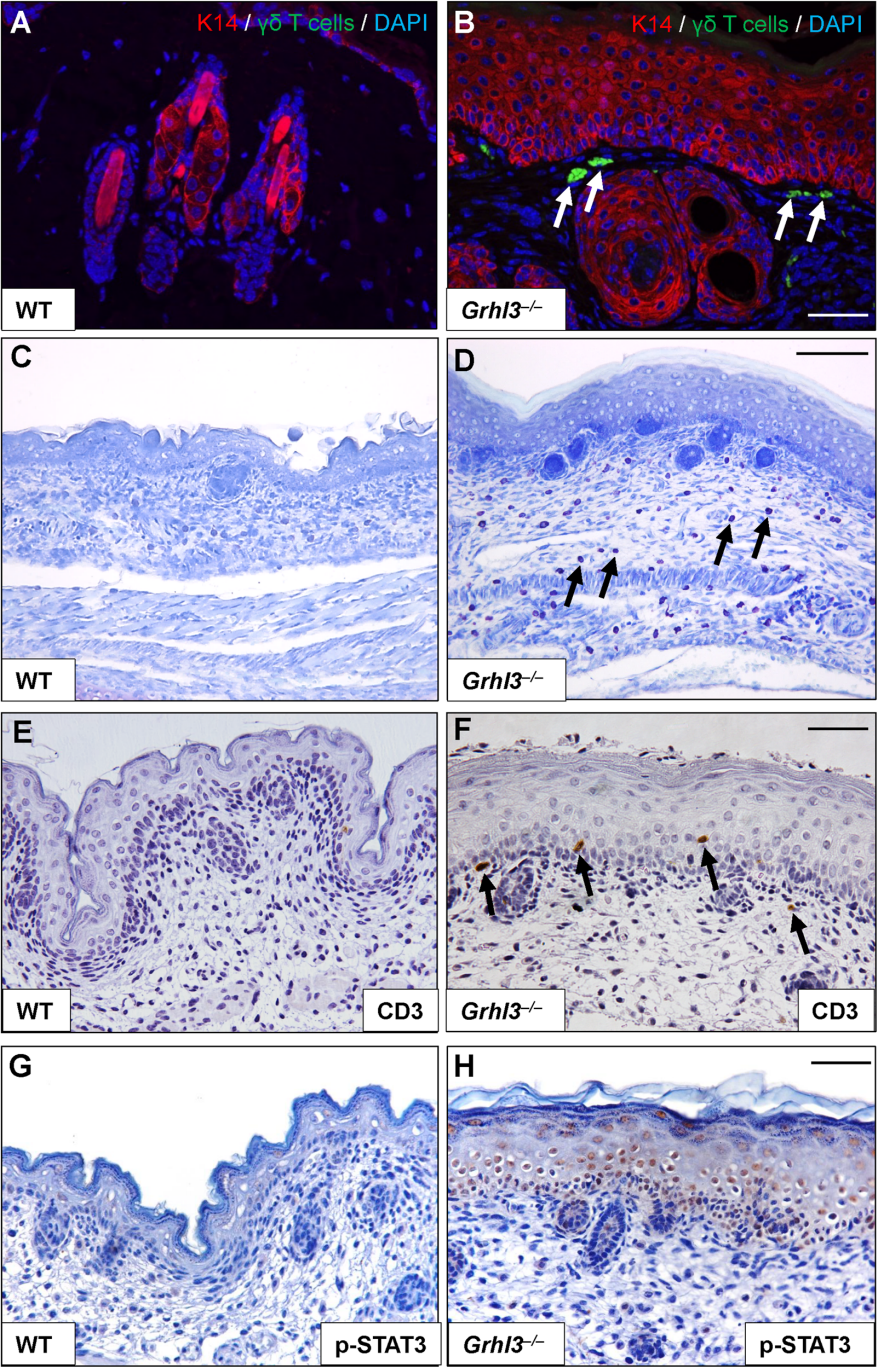
*Grhl3*^–/–^ embryonic skin shows compromised barrier integrity and increased expression of pro-proliferative cytokines. Infiltrating γδT cells (**a**, **b**; immunohistochemistry; arrows. Scale bars correspond to 50 μm) and elevated numbers of mast cells (**c**, **d**) are seen in the skin of *Grhl3*^–/–^ but not WT at E18.5, indicative of compromised epidermal integrity. Examination of the T-cell marker CD3 (**e**, **f**) shows increased expression in the dermis and epidermis of E18.5 *Grhl3*^–/–^ embryos associated with epidermal activation of the inflammatory/proliferative marker STAT3 (**g**, **h**). Scale bars correspond to 70 μm

**Fig. 3:**
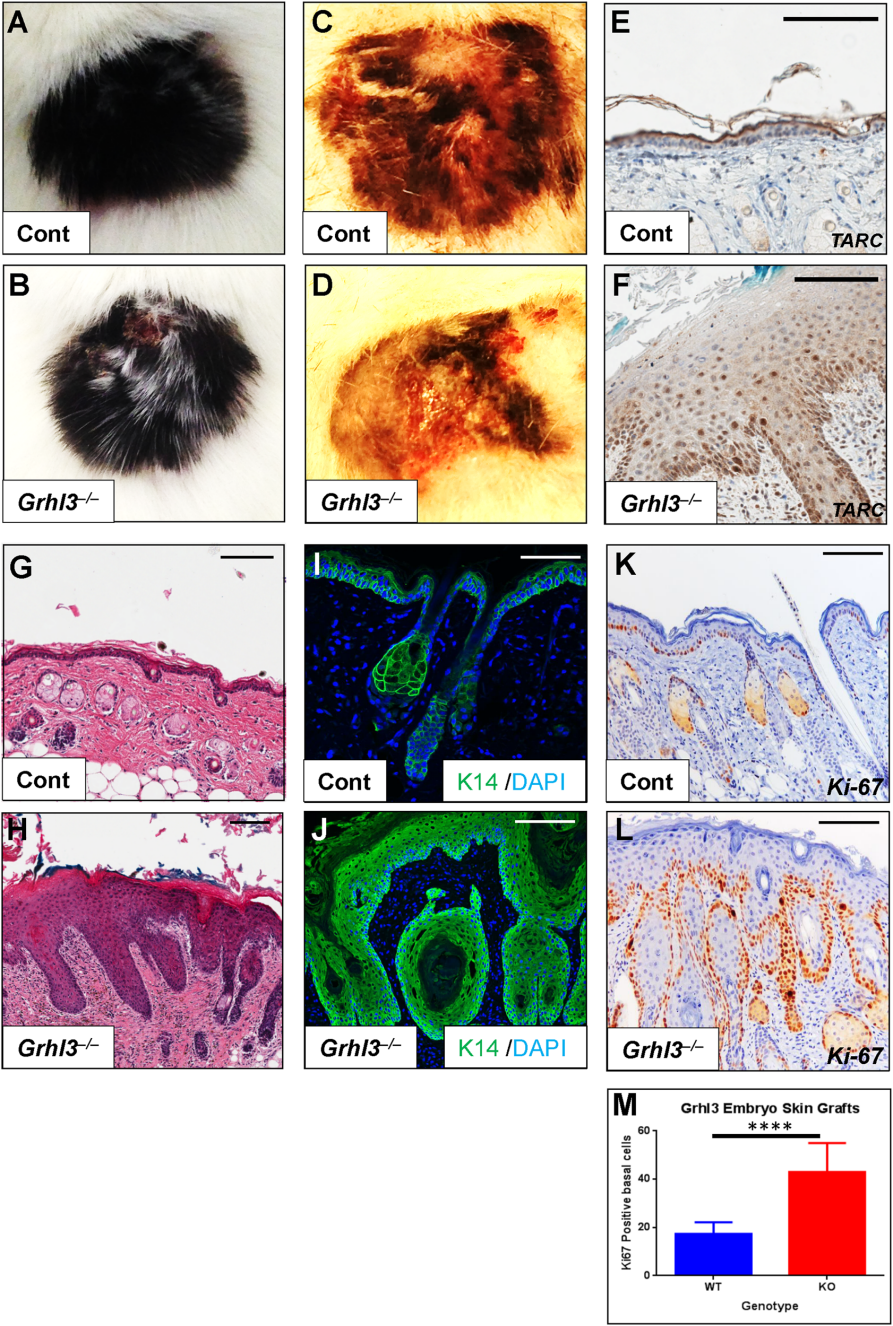
*Grhl3*^*–/–*^ embryonic skin presents with barrier defects, elevated TARC and hyperproliferation in the absence of systemic immune response. E18.5 back skin from (black) WT and Grhl3–/– embryos was grafted onto the back of (white) adult NOD-SCID-gamma (NSG) mice and grown for 4 months (**a**, **b**). Skin from *Grhl3*^–/–^ embryos presented with a patchy, scaly red appearance (**b**, **d**) as compared with WT skin (**a**, **c**). When these grafts were processed histologically at 4 months, they showed redistribution of TARC (**e**, **f**), and were found to be highly proliferative (**g**, **h**), resembling keratoacanthoma and well-differentiated SCC. We also noted a significant elevation in the expression of Keratin 14 (K14) (hyperkeratinisation) in *Grhl3*^*–/–*^ skin compared with WT (**i**, **j**). Expression of the proliferation marker Ki-67 is significantly increased in both the basal and suprabasal layers of the grafts from *Grhl3*^–/–^ skin compared to WT (**k**, **l**), confirmed by quantitation (**m**). **** indicates *p* < 0.001. All scale bars correspond to 100 μm

As TARC is a potent pro-inflammatory and pro-proliferative cytokine, and having identified TARC upregulation following abrogation of *Grhl3* in both human cultured keratinocytes, and within the skin of E18.5 *Grhl3*^–/–^ embryos, we next examined *Grhl3*^–/–^ embryonic skin for signs of a heightened immune and/or inflammatory response. We noted that the abundance of T cells (γδT cells) and granulated mast cells were both increased in the skin of *Grhl3*^–/–^ E18.5 mouse embryos compared with WT littermate controls (Figs. 2a–d). Similarly, we examined the expression of T-cell marker CD3 (Figs. 2e, f) and Signal Transducer and Activator of Transcription 3 (STAT3) transcription factor (Figs. 2g, h), and again found stronger in expression in *Grhl3*^–/–^ E18.5 mouse embryo skin. The quantifications of γδT cells (Fig. S4A) and positive CD3 cells (Fig. S4B) showed significant differences between *Grhl3*^–/–^ and WT skin. Taken together, these data suggest the existence of a pro-inflammatory and/or pro-proliferative microenvironment in *Grhl3*^–/–^ E18.5 mouse embryonic skin. A critical question therefore was whether the barrier defects and hyperproliferation seen in the skin of *Grhl3*^–/–^ embryos were caused due to production of keratinocyte/skin-intrinsic factors, or whether this was a secondary defect of systemic *Grhl3* deletion. To answer this question, back skin from WT or *Grhl3*^–/–^ embryos was grafted onto the back of adult NSG mice (Figs. 3a, b), which are severely immunocompromised and lack any inflammatory response. Interestingly, we found that even in the absence of systemic *Grhl3* deletion, the skin grafts manifest phenotypic signs of inflammation (Figs. 3b, d) as compared with the WT control skin (Figs. 3a, c), indicative of a cell-intrinsic pro-inflammatory cytokine production. This mirrors the effect of GRHL3 inhibition in cultured HaCaT cells and the cell-intrinsic overexpression of TARC. Grossly, the skin developed areas of hyperkeratinisation, alopecia and superficial ulcerations. After growing for 4 months, mice were euthanized and the grafts isolated and processed histologically. Consistent with prior reports^17^, TARC was found as a granular layer band in grafted WT skin. TARC expression, however, acquired a distinctly different profile in hyperproliferative, air-exposed grafted *Grhl3* KO skins when compared against both WT grafted skins and E18.5 *Grhl3* KO skins (Figs. 3e, f). Instead of a predominant expression in differentiating keratinocytes, TARC detection redistributed to basal keratinocytes. Skin from *Grhl3*^–/–^ grafts was hypercellular and parakeratotic with no visible granular layer (Figs. 3g, h). *Grhl3*^–/–^ grafts further presented with strong hyperkeratinisation with expansion of Keratin 14 above the basal layer (Figs. 3i, j), both apparent hallmarks of papilloma or pre-malignant SCC. An examination of cellular proliferation by Ki-67 staining similarly showed a significant increase in proliferating cells within the basal layer of the epidermis of *Grhl3*^–/–^ grafts (Fig. 3k–m), consistent with previously reported findings^11^. Taken together, these data suggest that the defective barrier integrity in *Grhl3*^–/–^ embryos is not mediated by the host immune response but rather, the keratinocytes themselves present with defective signalling, which ultimately causes barrier dysfunction, similar to that seen in Harlequin Ichthyosis^6^.

### Suppression of inflammation through the inhibitor 5ASA rescues epidermal structure, integrity and barrier protein expression in *Grhl3*^–/–^ embryo skin

**Fig. 4:**
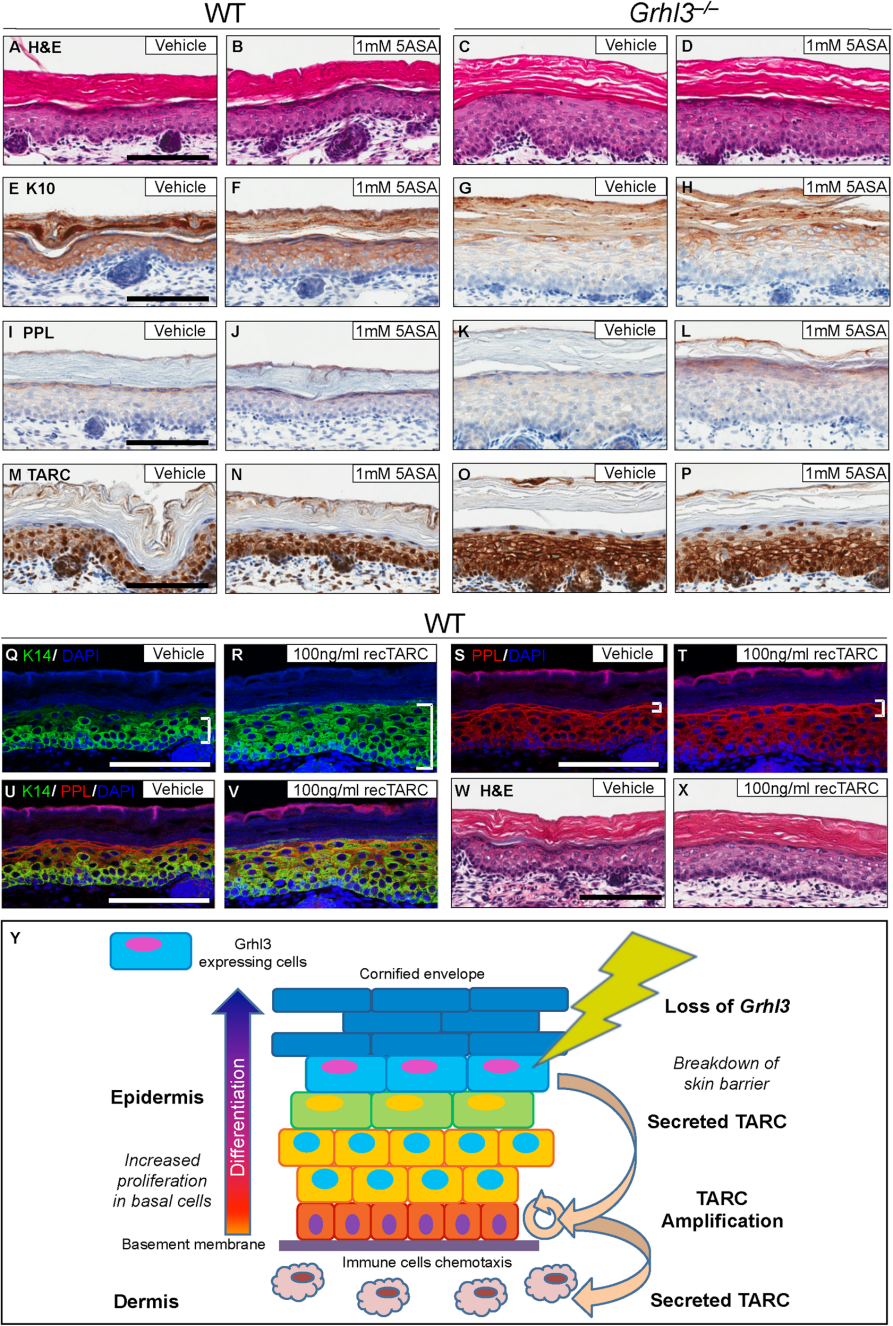
Compromised epidermal integrity in *Grhl3*^*–/–*^ skin is improved through application of 5ASA. Back skins from E16.5 WT and *Grhl3*^–/–^ embryo were cultured together with vehicle control (**a**, **c**) or in the presence of 1 mM *5ASA* (**b**, **d**). No significant changes in WT epidermal morphology were observed following 5ASA treatment of WT skin, however, treatment of the *Grhl3*^–/–^ skin (**c**, **d**) restored granular layer formation. The epidermal differentiation marker, K10, was also specifically increased in *Grhl3*^–/–^ skins following *5ASA* treatment (**e**-**h**), and expression of the structural barrier integrity protein PPL (**i**-**l**) was restored. 5ASA did not affect the skin from WT animals (**m**, **n**) but reduced the expression of TARC in *Grhl3*^*–/–*^ skin (**o**, **p**). The treatment of E16.5 WT skin with recombinant TARC demonstrates expansion of the basal layer Keratin 14-positive compartment and reduced keratinocyte compaction towards the granular layer (**q**-**v**). The granular layer is less pronounced in H&Es (**w**, **x**). Scale bars correspond to 100 μm. These data are summarised in the proposed functional model (**y**) suggesting that loss of *GRHL3* in differentiated cells leads to a compromised epidermal barrier with concomitant TARC production and secretion to stimulate proliferation of basal keratinocytes. This triggers additional TARC expression from basal cells amplifying the proliferative signal

We next set out to determine whether abrogation of TARC signalling could rescue barrier formation, maintenance and integrity. In the absence of specific TARC inhibitors, we used the broad spectrum anti-inflammatory 5ASA/mesalamine, which is reported to inhibit both Tumor Necrosis Factor-alpha (TNF-α) and Interferon gamma (IFNγ), which are in turn key regulators of TARC expression^18–20^. We treated E16.5 embryo back skin cultures with *5ASA* for 4 days. Back skin from WT control animals showed normal stratified squamous keratinising epithelium following both vehicle and 5ASA treatment, with identifiable layers of progressive differentiation, as cells migrated from their attachments at the basement membrane to the outer cornified envelope (Figs. 4a, b). Vehicle-treated *Grhl3*^–/–^ embryo back skin presented with overall thickening with increased cellularity and disruption of the stratum granulosum. Conversely, following 5ASA treatment, *Grhl3*^–/–^ back skin showed improvement in formation of the stratum granulosum (Figs. 4c, d). Furthermore, expression of the epidermal differentiation marker Keratin 10 (K10) (Fig. 4e–h) is significantly increased, and expression of the barrier protein Periplakin (PPL) (Fig. 4i–l) is largely restored. Interestingly, embryonic WT skins when cultured ex vivo exhibited upregulated TARC expression relative to freshly isolated embryonic skins, such that most basal and suprabasal keratinocyte nuclei became TARC positive. On this background, *Grhl3*^*–/–*^ skin showed further elevated expression such that TARC now also accumulated in the cytoplasm and cell membranes of most keratinocytes with the exception of those just beneath the cornified envelope. 5ASA treatment slightly reduced TARC immunoreactivity in *Grhl3*^*–/–*^ skin, however, no difference was observed in WT skin (Fig. 4m–p and Fig. S5A). To examine the direct effects of TARC on keratinocyte proliferation and differentiation, we next cultured WT mouse embryonic skins ex vivo with recombinant TARC. TARC-treated skins showed expansion of the epidermal basal compartment reflected by an increase in K14 expressing cells, as well as a reduction in keratinocyte compaction towards the PPL^high^ granular layer. The granular was also less pronounced by Hematoxylin and Eosin (H&E) staining (Fig. 4q–x). These data confirm that TARC-mediated inflammation promotes basal cell expansion and inversely correlates with barrier integrity and differentiation in the skin of *Grhl3*^–/–^ embryonic mice. In addition, the proliferative potential induced by knocking down *GRHL3* in human keratinocytes was significantly reduced after treatment with 2 mM of *5ASA* for 48 h (Fig. S5B).

In summary, our data suggest that loss of *Grhl3* in suprabasal epidermal cells results in a breakdown of skin barrier integrity, release of the pro-proliferative cytokine TARC, and subsequently increased keratinocyte proliferation and loss of proliferative control in the basal population of epidermal stem and progenitor cells (Fig. 4y). 5ASA inhibition of inflammation and presumably TARC, leads to improved epidermal structural integrity following loss of *Grhl3*.

## Discussion

Although a number of studies have addressed the role of GRHL3 in the maintenance of skin barrier integrity, function and epidermal proliferation, our study is the first to identify a novel link between GRHL3 and a secreted, pro-proliferative signal. Our study provides evidence to explain how deletion of GRHL3, a transcription factor expressed exclusively within the suprabasal layers of the epidermis, can lead to enhanced proliferation, and impaired differentiation of the basal keratinocyte layer.

We demonstrated that compromised skin barrier formation due to *GRHL3* deletion stimulates expression of TARC, also known as CC-motif ligand 17 (CCL17)^21^, which promotes Th_2_ lymphocyte recruitment and epidermal hyperplasia. TARC is a secreted cytokine, belonging to the CC chemokine family, and is primarily expressed in the thymus and within the peripheral circulation in mononuclear cells. TARC elicits its function through binding the CCR4 receptor, leading to chemotaxis when activating the receptor on T cells^22^, and platelet activation and clotting when stimulating this receptor on platelets^23,24^. TARC is also significantly associated with hyperproliferative disorders including various lymphomas^25,26^, hepatocellular carcinoma^27^, cervical^28^ and gastric^29^ cancers, as well as numerous disorders of the skin, particularly AD^16,30,31^, alopecia areata^32^ and pigmented purpuric dermatitis^33^. A growing body of evidence suggests that TARC plays a role in several inflammatory skin diseases including AD, psoriasis and mycosis fungoides (a cutaneous T-cell lymphoma)^34–36^. Studies in mice and humans have demonstrated that keratinocyte expression of TARC is increased as AD lesions develop^5,37^. Furthermore, elevated levels of TARC have also been observed in the serum of patients with AD^37^ and recently TARC has been utilised as a clinical biomarker for AD^5^. However, to date, TARC has not been identified as a pro-proliferative cytokine for the regulation of keratinocyte proliferation and differentiation^35^.

Our data demonstrate that KD of *GRHL3* in human keratinocytes induced an inflammatory response through the secretion of TARC, resulting in increased proliferation within individual clones, without affecting the total number of colonies generated. These data suggest a potent pro-proliferative impact of TARC on keratinocytes as a consequence of abrogated GRHL3 signalling. From immunohistochemistry analysis of freshly isolated WT embryonic and grafted skin, TARC is predominantly expressed by keratinocytes of the granular layer (or just under the granular layer), much like GRHL3. However, following promoter analysis of the TARC/CCL17 gene, we were unable to find a conserved transcriptional binding site for GRHL3. In utero embryonic skins of both WT and KOs show weaker TARC detection in basal cells, which is absent in WT grafted skin, suggesting basal detection of TARC correlates with proliferative status. Given TARC is most prominent in late differentiating keratinocytes, we contend that in vivo TARC originates from granular layer (and near granular layer cells). When the barrier is immature or upon breakdown, TARC is secreted to act in a paracrine manner to stimulate basal layer keratinocyte proliferation and amplifies the process by promoting additional TARC expression from basal cells. Once basal keratinocyte TARC expression initiates, the process would be self-sustaining even when granular layer formation fails during transition to parakeratosis. Secreted TARC would also act as a potent chemokine to attract γδT cells and promote mast cell differentiation (Fig. 4y). Future studies will be directed toward generating a double knockout of *Grhl3* and *TARC* mouse model to potentially rescue the *Grhl3*^*–/–*^ phenotype including the pro-clotting effect. Interestingly, inhibiting the TARC-mediated response with 5ASA led to partial mitigation of the abnormal differentiation and architecture seen in vehicle-treated *Grhl3*^*–/–*^ compared with WT skin. 5ASA, also known as mesalamine/mesalazine, is a disease modifying aminosalicylate anti-inflammatory drug used in the management of inflammatory bowel conditions, such as ulcerative colitis and Crohn’s disease. These findings suggest that GRHL3 may play a pivotal role in suppressing the expression of TARC to prevent immune-mediated epidermal hyperplasia.

The role of inflammatory mediators in skin disorders has been well described, but more specifically the effects of deleting *GRHL3* in skin has been linked to the initiation and progression of hyperproliferative skin conditions such as psoriasis by interleukin mediated T-cell activation^13^. The authors predicted interleukin targets by microanalysis of skin from the K14cre-*Grhl3* mouse, in contrast to our analyses of secreted protein. Of note, the injured K14cre-*Grhl3* skin presented by Gordon et al. ^13^, closely resembles our findings of the grafted *Grhl3* embryo skin.

The concept of inflammatory cytokines stimulating hyperproliferation and even stimulating neoplastic lesions has been explored in other skin models. Wounding of the InvEE mouse model stimulates a release of IL-1α resulting in the infiltration of immune cells, causing a pro-tumorigenic inflammatory environment. Neoplastic tumours developed upon wounding these mice, but the lesions could be prevented by pharmacologically blocking the IL-1α-mediated response^38^. Prior to this, the importance of specific cytokine and inflammatory cell interactions had been shown in the same mouse model. Bone marrow rescue, using marrow from γδT-cell^–/–^ donor mice, showed an almost complete lack of wound-induced tumour formation. This was similar to results where the mouse immune system was heavily suppressed with pharmacological agents such as dexamethasone or cyclosporin A. In combination, these data support our findings that an inflammatory response stimulated by deletion of a single gene, i.e., *GRHL3*, and thereby causing secretion of TARC by keratinocytes would be sufficient to generate a hyperproliferative response, and perturb the normal differentiation programing of the epidermis.

Further investigations on the role of GRHL3 in inflammation and hyperproliferation would logically progress to skin carcinogenesis experiments with 5ASA and other specific bioavailable inhibitors to determine whether inhibiting the TARC inflammatory pathway may impact on the initiation or development of skin tumours.

## Conclusion

Deletion of the transcription factor GRHL3 in the skin initiates a chemokine responsive signal leading to keratinocytes hyperproliferation and a pro-tumorigenic state. We identified TARC/CCL17 as a primary mediator in this pathway, and have shown that an anti-inflammatory drug, 5ASA, can partially restore epidermal differentiation, ex vivo.

## Electronic supplementary material

Figure S1

Figure S2

Figure S3

Figure S4

Figure S5

Supplementary Figure Legends
