# Supplementary figures and images for: Loss of GRHL3 leads to TARC/CCL17-mediated keratinocyte proliferation in the epidermis
Source: Cell Death Dis. 2018 Oct 19;9(11):1072. doi: 10.1038/s41419-018-0901-6 (PMC6195598; doi:10.1038/s41419-018-0901-6)

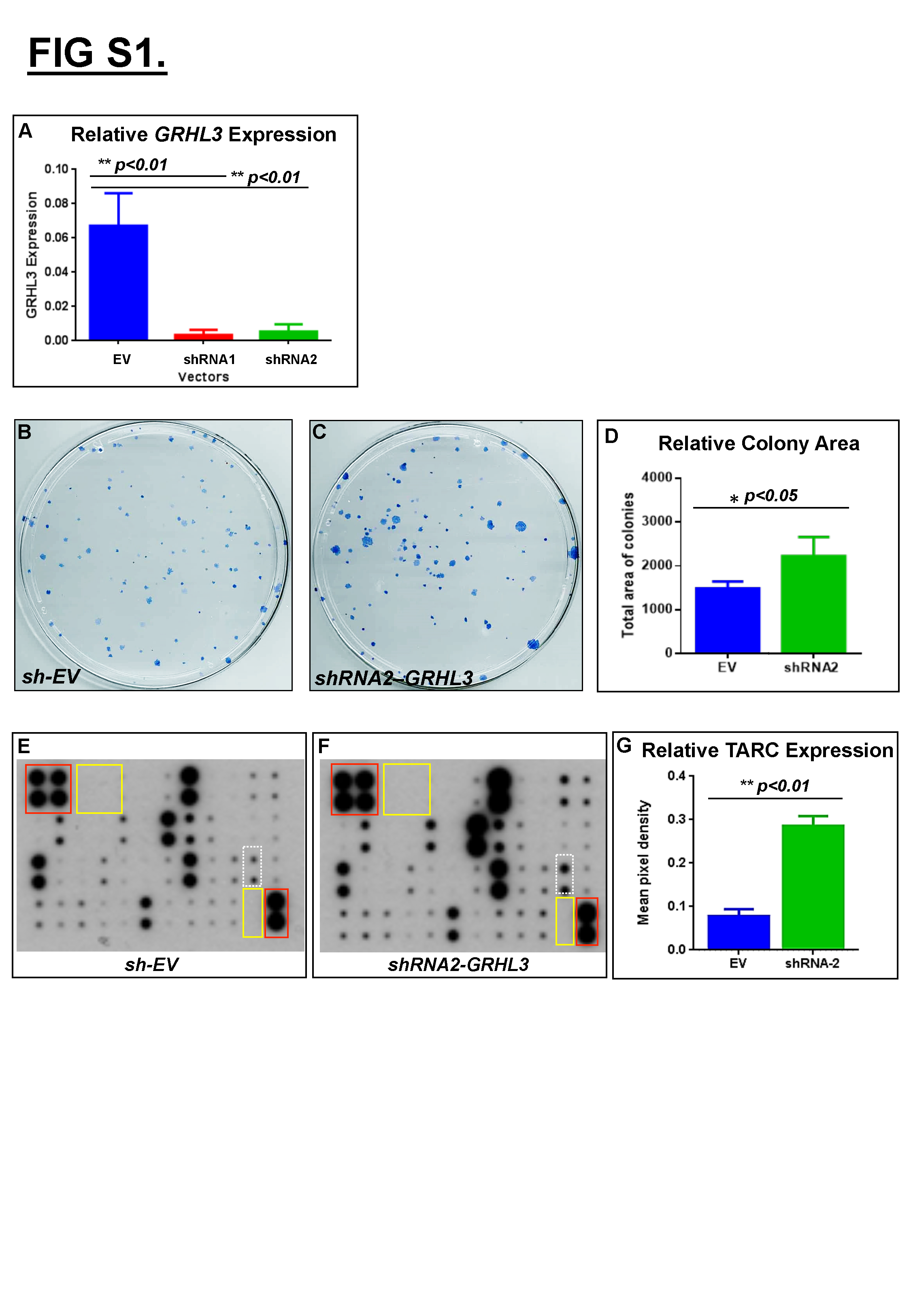

Supplement: Supplementary file 1 — Figure S1 [file 41419_2018_901_MOESM1_ESM.tif]

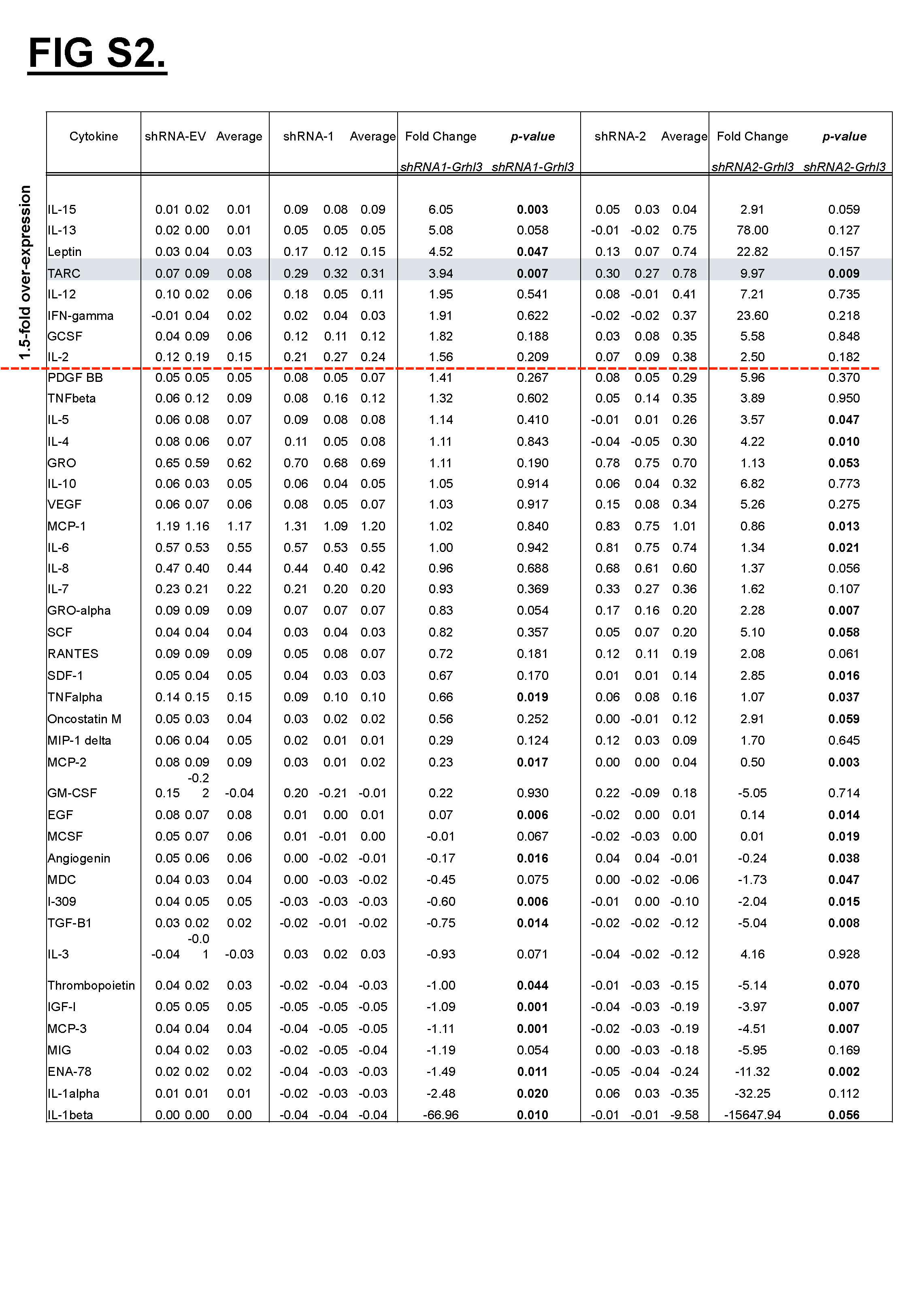

Supplement: Supplementary file 2 — Figure S2 [file 41419_2018_901_MOESM2_ESM.tif]

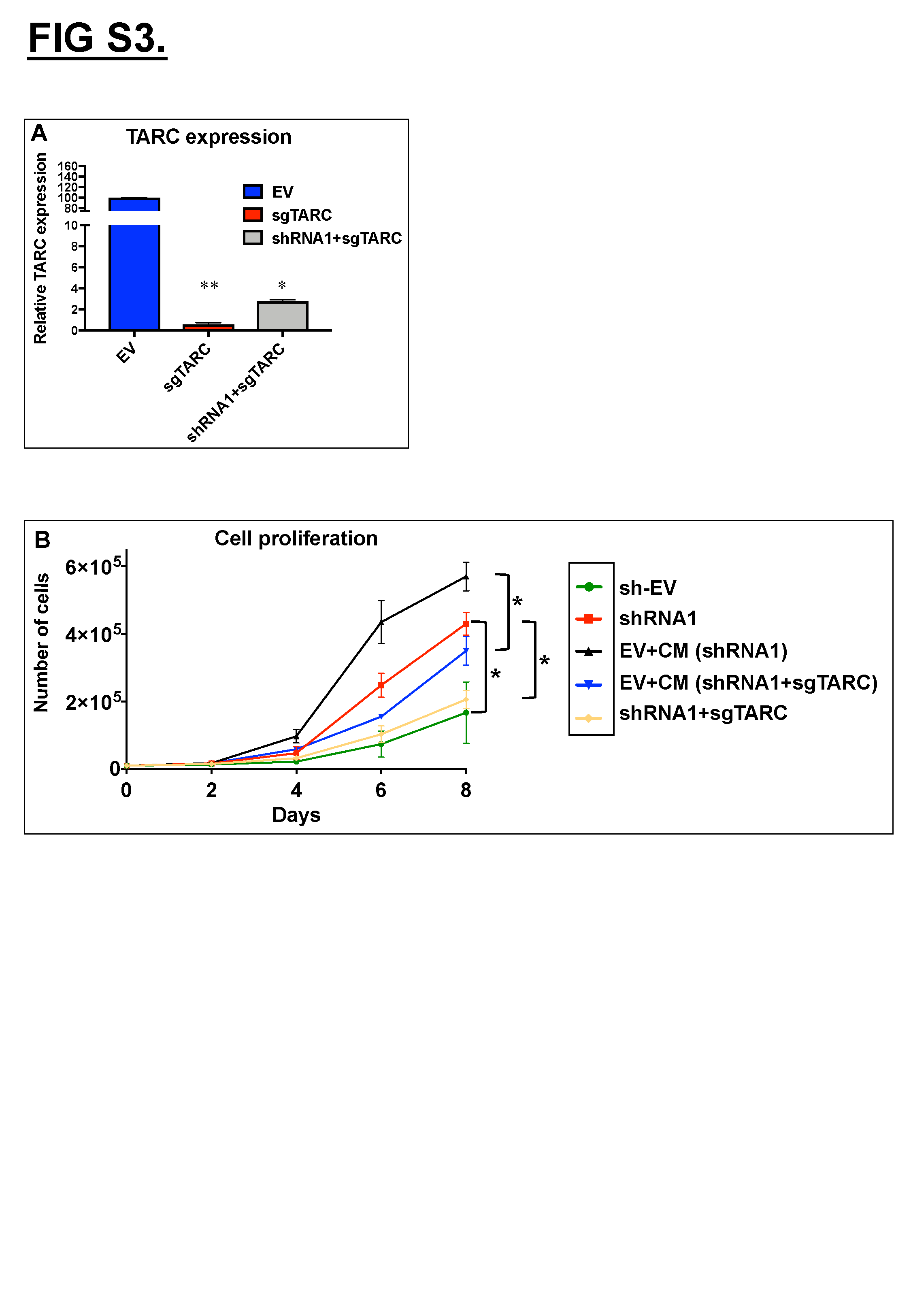

Supplement: Supplementary file 3 — Figure S3 [file 41419_2018_901_MOESM3_ESM.tif]

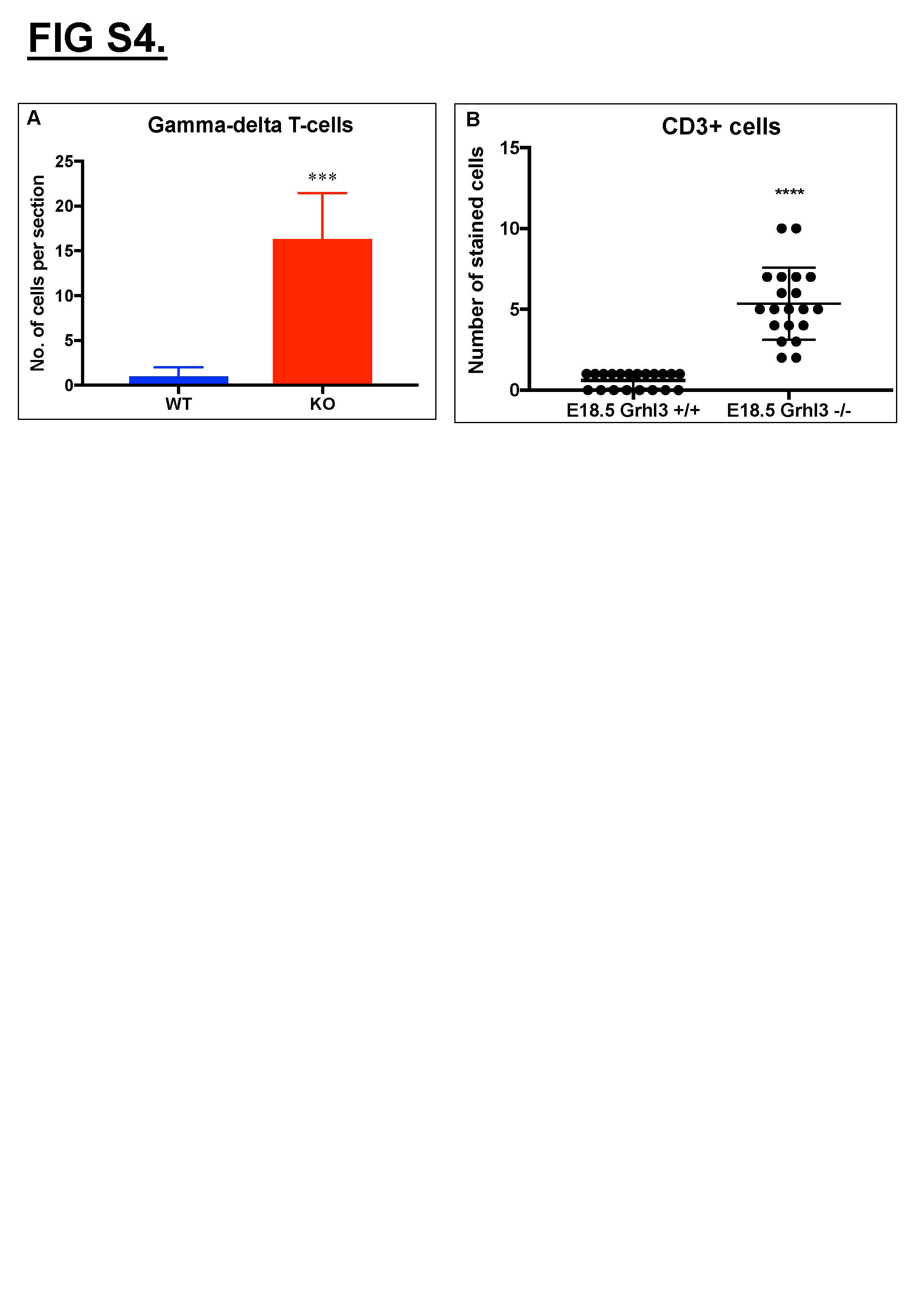

Supplement: Supplementary file 4 — Figure S4 [file 41419_2018_901_MOESM4_ESM.tif]

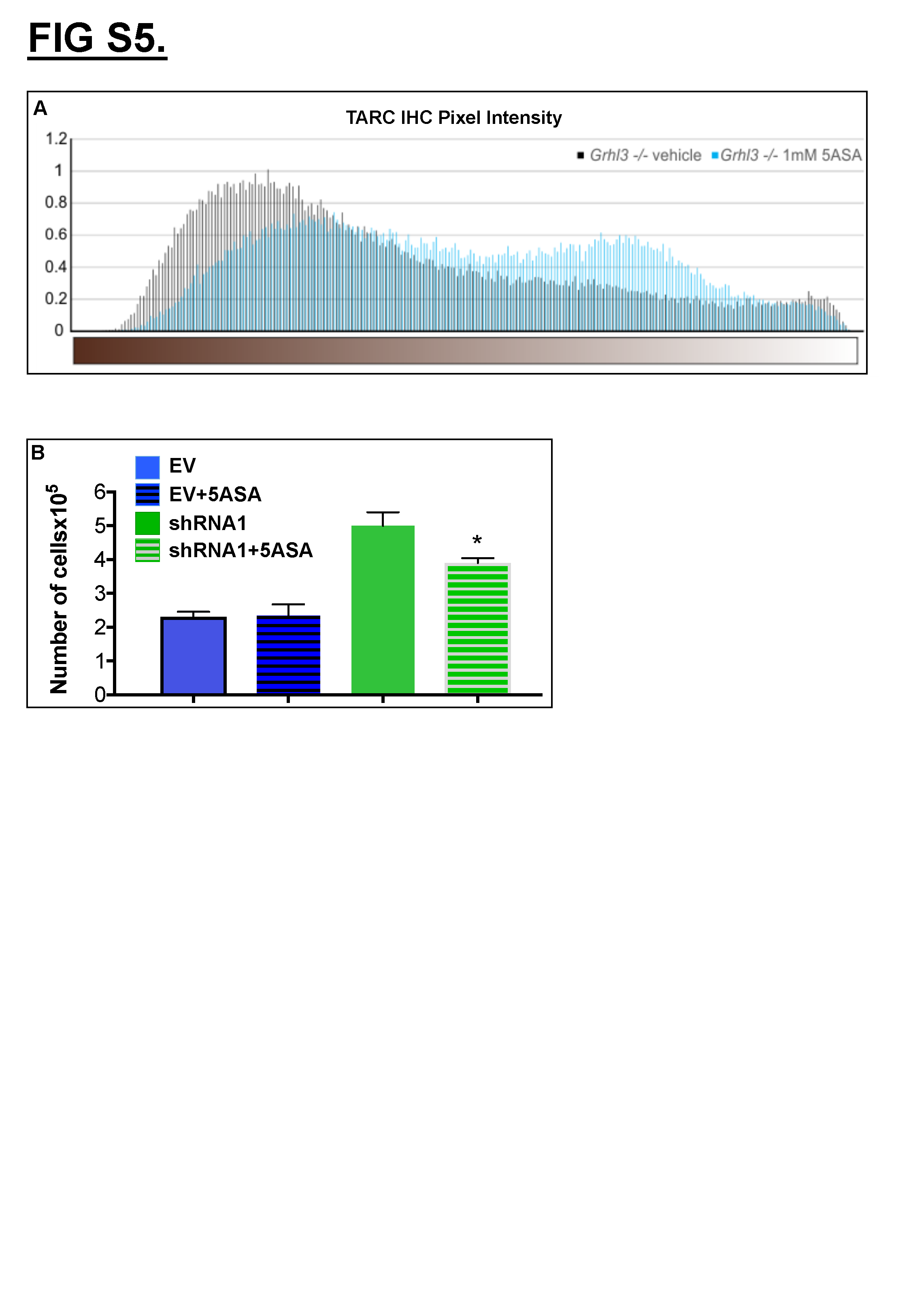

Supplement: Supplementary file 5 — Figure S5 [file 41419_2018_901_MOESM5_ESM.tif]
