# Supplementary material for: Loss of GRHL3 leads to TARC/CCL17-mediated keratinocyte proliferation in the epidermis
Source: Cell Death Dis. 2018 Oct 19;9(11):1072. doi: 10.1038/s41419-018-0901-6 (PMC6195598; doi:10.1038/s41419-018-0901-6)
Supplement: Supplementary file 6 — Supplementary Figure Legends [file 41419_2018_901_MOESM6_ESM.docx]

**Supplementary Figure Legends**

**Fig. S1. Loss of *GRHL3* (via knockdown with shRNA-2) also leads to increased keratinocyte cell proliferation and elevated TARC expression.** Transduction of HaCaTs with either of two shRNAs targeting *GRHL3* (shRNA-1 and shRNA-2 respectively) leads to significant reduction in abundance of *GRHL3* mRNA (A). KD of *GRHL3* via shRNA (*shRNA2*) leads to increased colony size (B-C), relative to transduction with empty vector (*sh-EV*), albeit not increased colony number (not shown) in the human epidermal keratinocytes, a finding confirmed by quantitation of total colony area per plate (D). Analysis of cytokine activity in conditioned medium (CM) collected from HaCaT cells transduced with *sh-EV* (E) or *shRNA2-GRHL3* (F) show that as for *shRNA1-GRHL3,* the only cytokine which is significantly overexpressed (when quantitated by densitometric scanning; (G) following *GRHL3* KD is TARC (white box). Positive (red boxes) and negative controls (yellow boxes) are also shown. Control *sh-EV* data repeated from Fig 1. * and ** represents p<0.05 and p<0.01, respectively.

**Fig. S2. Cytokine expression following *GRHL3* knockdown in human keratinocytes.** Table of cytokine expression (quantitated by densitometry) in CM from HaCaTs transduced with either of two *shRNAs* targeting *GRHL3* (*shRNA-1* and *shRNA-2* respectively), relative to HaCaT cells transduced with *shRNA-EV*. Of cytokines upregulated at least 1.5-fold following KD (dashed line), the only cytokine, which is significantly (p<0.05) upregulated in both treatments is TARC (grey box).

**Fig. S3. Deletion of *TARC* in *shRNA1-Grhl3* HaCaT cells reduced cell proliferation.** (A) CRISPR-Cas9 mediated deletion of *TARC* was confirmed by qPCR showing loss of *TARC* expression in sgTARC and double shRNA1+sgTARC in comparison to EV cells. (B) KD of *GRHL3* (shRNA1) increased cellular proliferation in comparison to control EV cells (* and ** indicates p<0.05 and p<0.01, respectively). The Dox-inducible deletion of *TARC* significantly reversed the hyperproliferative phenotype in shRNA1-Grhl3 cells to a proliferation rate similar to EV cells (p<0.05). CM from double knockdown shRNA1+sgTARC cells did not stimulate EV proliferation to the same extent as CM from shRNA1 cells (p<0.05).

**Fig. S4. Quantification of Gamma-delta T-cells and CD3 immunostaining.** The quantifications of (A) γδT-cells as presented in Fig. 2A-B and (B) CD3 positive cells as shown in Fig. 2E-F, represent the relative number of cells per section (n=20) between 3 WT and 3 Grhl3^–/–^ E18.5 skin. Statistical significance is presented as *** p<0.001 (A) and **** p<0.0001 (B).

**Fig. S5. 5ASA treatment of *GRHL3*-KD cells decreased cellular proliferation.** (A) TARC IHC pixel intensity measured using ImageJ shows lower intensity of TARC staining following 1mM of *5ASA* treatment of Grhl3^–/–^ skin. Results are representative of pair matched *Grhl3^–/–^* vehicle and 5ASA-treated *ex vivo* cultured skins from 5 *Grhl3^–/–^* embryos. (B) Treatment of *sh-EV* and *GRHL3-KD* HaCaT cells with *5ASA* for 48 hours led to a significant decrease (* p<0.05) in the cell number of KD cells with no effect on EV cells.
